# Safety and effective use of landiolol in the ICU

**DOI:** 10.1186/2052-0492-2-16

**Published:** 2014-02-21

**Authors:** Toru Hifumi, Hiroshi Kato, Yuichi Koido, Kenya Kawakita, Yasuhiro Kuroda

**Affiliations:** Emergency Medical Center, Kagawa University Hospital, 1750-1 Ikenobe, Miki, Kita, Kagawa, 761-0793 Japan; Division of Critical Care Medicine and Trauma, National Hospital Organization Disaster Medical Center, 3256 Midori-cho, Tachikawa, Tokyo, 190-0014 Japan

**Keywords:** Landiolol, Beta-blocker, Cardiovascular response, Endotracheal suctioning, Close monitoring

## Abstract

Supplemental landiolol administration (20 or 40 μg kg^-1^ min^-1^) effectively diminished harmful hemodynamic changes during bronchoscopic endotracheal suctioning compared to normal saline. However, inappropriate use of landiolol (i.e., failure of evaluating factors that influence hemodynamic changes) may iatrogenically further complicate pathophysiology, and relatively higher doses of landiolol may be dangerous. We recommend that landiolol should not be routinely used to control cardiovascular responses during bronchoscopic endotracheal suctioning in the intensive care unit. Careful evaluation of factors influencing hemodynamic changes and close monitoring of the patient are mandatory following landiolol administration. Furthermore, a lower initiation dose is recommended.

## Correspondence/Findings

We read the article *Landiolol reduces hemodynamic responses to bronchoscopy-assisted suctioning in intubated ICU patients* by Tochikubo et al. with great interest [1]. The authors prospectively conducted a double-blind control study using a cross-over design to evaluate the protective effects of landiolol against cardiovascular responses during bronchoscopic endotracheal suctioning. Because there is a word limitation for publishing the current manuscript as a *Letter to the Editor*, not *Original Article*, we assume that authors were not able to provide sufficient data. Therefore, we would like to clarify the details of the current great literature through this letter.

We recently reported our clinical experience with the use of landiolol hydrochloride for conservative management of blunt aortic injury (BAI) [2]. In BAI patients, aggressive blood pressure management along with decreased heart rate and contractility with beta-blocker therapy is critical for decreasing aortic wall tension, and it is essential during nonoperative management or during the treatment period leading up to delayed surgery. Heart rate is influenced by several factors such as hypovolemia due to massive bleeding, pain, anxiety, fever, and sympathetic activation in trauma patients. Therefore, we not only clarified the significance of using landiolol but also emphasized that careful evaluation of factors influencing hemodynamic changes and close monitoring of vital signs are mandatory during beta-blocker administration.

Although details regarding the patient’s baseline characteristics remain unknown, it appeared that Tochikubo et al. [1]. did not adjust several factors that influence hemodynamic changes such as hypovolemia, pain, fever, and sympathetic activation in each group. We are greatly concerned that the inappropriate use of landiolol (i.e., failure of evaluating factors that influence hemodynamic changes) may iatrogenically further complicate patient’s pathophysiology.

Tochikubo et al. [1] mentioned that endotracheal suctioning stimulus induces undesirable cardiovascular sympathetic responses and cited literature conducted by Jongerden et al. [3]; however, Jongerden et al. concluded that changes in heart rate, mean arterial pressure, and oxygen saturation were mild both during and after use of closed and open suction systems without the administration of landiolol [3]. When control of cardiovascular responses during bronchoscopic endotracheal suctioning is required, transient deeper sedation and analgesia including local anesthesia in the trachea during the procedure, instead of administering beta-blockers, is considered a simple first-line strategy.

Moreover, additional indications of landiolol use for tachycardia in ICU patients have been recently approved by the Ministry of Health, Labour, and Welfare [4]; therefore, the Japanese Circulation Society published a statement for the appropriate use of landiolol [5]. These guidelines recommend initiation with a lower dose of landiolol (1 μg kg^-1^ min^-1^) for patients with impaired left ventricular function because the clinical safety for those who severely impaired left ventricular function has not been evaluated. Tochikubo et al. used 20 or 40 μg kg^-1^ min^-1^ landiolol to control cardiovascular responses during bronchoscopic endotracheal suctioning in the current study; therefore, we were concerned regarding the safety of landiolol use at these dosages. In fact, after bronchoscopic endotracheal suctioning, patients in the landiolol groups exhibited decreased heart rate and systolic blood pressure from baseline. Although patients with severely impaired left ventricular function appeared to be excluded in the current study, we recommend that they provide the inclusion and exclusion criteria.

In conclusion, we recommend that landiolol should not be routinely used for control of cardiovascular responses during bronchoscopic endotracheal suctioning in the ICU patients. Careful evaluation of factors that influence hemodynamic changes and close monitoring of vital signs are mandatory during landiolol administration. In addition, a lower initiation dose is recommended.
